# MOUD 2.0: a clinical algorithm and implementation evaluation protocol for sublingual and injectable buprenorphine treatment of opioid use disorder

**DOI:** 10.3389/fpsyt.2024.1383695

**Published:** 2025-01-21

**Authors:** Brandon L. Joa, Eric N. Fung, Michael S. Weinstein, Lara C. Weinstein

**Affiliations:** ^1^ Sidney Kimmel Medical College, Thomas Jefferson University, Philadelphia, PA, United States; ^2^ Department of Epidemiology, Harvard T.H. Chan School of Public Health, Boston, MA, United States; ^3^ Integrated Care Clinic, Project HOME Healthcare Services, Philadelphia, PA, United States

**Keywords:** MOUD, low-barrier, underserved medicine, harm reduction, substance use disorder

## Abstract

**Background:**

Primary care is the initial contact point for most patients with opioid use disorder (OUD) but lacks tools for guiding treatment. Only a small fraction of patients access evidence-based care. Long-acting injectable buprenorphine has potential to improve medication adherence and program retention in low-barrier primary care treatment settings. We present the first clinical decision support algorithm incorporating long-acting buprenorphine (LAIB) in primary care. We include a protocol for a future evaluation of the algorithm’s implementation process, “Medication for Opioid Use Disorder (MOUD) 2.0,” at a housing and integrated care clinic at a Federally Qualified Health Center.

**Methods:**

Literature review and expert consensus informed creation of the algorithm, which underwent iterative development with feedback from clinicians, staff, and patients. Patients are categorized by adherence to therapy and retention in the program, with recommendations for each category. Adherence is determined by urine screen supplemented by self-report. To ensure all patients in this high morbidity and mortality risk population are treated, we will treat patients as their own controls in the evaluation, with potential for multisite comparisons. We will present descriptive statistics for adherence proportion before and after MOUD 2.0 implementation, testing for differences using McNemar’s test. We will then present pre- and post-implementation unadjusted six-month survival curves for retention.

**Discussion:**

LAIB is incorporated as an alternative or adjunctive treatment for patients refractory to sublingual buprenorphine and as an initial treatment for selected patients. We developed an algorithm with 4-, 8-, and 12-week decision points to guide treatment for patients with varying levels of response to sublingual buprenorphine and LAIB. This clinical decision tool incorporates LAIB among treatment options for OUD in primary care settings. The protocol will evaluate the algorithm’s implementation, presenting a replicable method for assessing adherence and retention among high-risk patients in similar settings.

## Background and rationale

The opioid epidemic accounts for high morbidity and mortality in the United States, contributing to 68,630 deaths in 2021 and 81,806 in 2022 (1–3). Social isolation (4) and declines in institutions of meaning and connection—e.g., family, religious communities, local and voluntary associations—contribute to increasing prevalence of opioid use disorder (OUD) and complications, leading to drops in life expectancy (5). Primary care is the first line of treatment for OUD in most communities. Yet, fewer than 25% of patients with OUD who interact with primary care receive highly effective medication for opioid use disorder (MOUD) (6). As many as 26-60% of motivated patients depending on population may fail to gain access to timely treatment with a clinician (7, 8). To address social and medical factors of this multifaceted epidemic, primary care needs clinical support tools and screens for an integrative approach.

Against increased mortality and availability of high-potency opioids, new treatment options such as long-acting injectable buprenorphine (LAIB) have emerged (9). Buprenorphine is a partial opioid agonist effective in preventing relapse, controlling withdrawal symptoms, and reducing overdose risk (10). Prescribed as a sublingual film or tablet, buprenorphine has seen increasing use in the primary care setting through practitioner education programs (11, 12). Buprenorphine has a long elimination half-life of 24-48 hours and reaches maximum serum concentration within 2 hours when taken sublingually, compared to around 12 hours to maximum concentration for LAIB [see (13)].

Yet, clinicians have recognized limitations to sublingual buprenorphine such as difficulty with adherence and diversion to non-clinical uses. Measuring adherence to buprenorphine relies on questionnaires alongside urine samples or buccal swabs, often generating equivocal results (14). In contrast to sublingual buprenorphine, as a new injectable route, LAIB can improve adherence while mitigating social factors associated with misuse and diversion (15). Among patients starting with fentanyl-positive urine in one randomized clinical trial, LAIB was associated with a significantly higher percentage of subsequent fentanyl-negative urine samples, compared to sublingual buprenorphine-naloxone (difference, 12.7%; 95% CI, 9.6%-15.9%) (16). Currently, injectable buprenorphine is much more expensive than sublingual buprenorphine. Judicious prescription thus balances potential benefits with costs to patients and clinics.

There are currently no guidelines for incorporating LAIB into stepwise treatment of OUD, so no algorithms and implementation studies yet exist. A PubMed search of the terms “MOUD, MAT, algorithm” in December 2023 yielded few results. Still, one description of a clinical algorithm (17) served as a good starting point for developing this study, in combination with the Substance Abuse and Mental Health Services Administration recommendations (18) and the American Society for Addiction Medicine practice guidelines (19). The previously proposed algorithm did not use results of drug testing, i.e. toxicology, to guide clinical decisions. Further, no studies to date included LAIB for clinical decision-making.

Filling these OUD treatment gaps and building on the aforementioned studies, treatment of OUD should ideally be fair, adaptable to patient response, and feasible within clinics’ resource limitations. To be perceived as fair, treatment must be consistent. Clinicians should consider objectively measurable and subjectively reported patient responses to treatment within a systematic framework, i.e., measurement based care (20, 21). We therefore developed a clinical decision support algorithm for treating OUD using measurement-based care in a primary care setting. This implementation study will evaluate the algorithm’s effects in an integrated primary and behavioral health setting. The following includes the implementation evaluation protocol followed by a full description of the algorithm.

## Methods

### Study objectives

The objective of this implementation evaluation is to assess whether the MOUD 2.0 algorithm improves treatment adherence and retention in the treatment program at the clinic-wide level, compared to these outcomes before implementation. The primary outcome measures are quantified adherence to MOUD and time length of retention in the treatment program.

Adherence will be operationalized as having a minimum of 3 buprenorphine appropriate drug screens (resulting positive for buprenorphine and norbuprenorphine) over a 6-month period regardless of substance use. Retention in treatment will be measured as the proportion of participants with at least 180 days of continuous pharmacotherapy with a medication prescribed for OUD, with no gaps greater than seven days (22). These outcome measures will show whether retention rates increase along with adherence, or whether the implementation favors one outcome measure over the other. The two measures are not mutually exclusive. In the context of an implementation evaluation, adherence could theoretically be more indicative of clinical response to the algorithm, whereas retention could characterize patient buy-in of the implementation. Secondary outcomes will include the percentage of patients starting LAIB for the first time, illicit use of substances per self-report and toxicology, and results of infectious disease while in the program.

### Evaluation design

The evaluation assesses the feasibility of implementing the MOUD 2.0 algorithm in primary care clinics through a retrospective chart review of clinical outcomes. Feasibility denotes advancing the algorithm’s objectives—improved adherence and retention—while maintaining patient access to consistent, measurement-based treatment. The algorithm is implemented at the clinic-wide level to preserve fairness between patients and clinicians, per the algorithm goal, as it would be unethical to exclude patients with OUD from treatment in the program. Therefore, structuring the evaluation as a retrospective implementation evaluation is more appropriate than as a randomized clinical trial to ensure full equal access to treatment.

The implementation evaluation will first be conducted at a Philadelphia Integrated Care Clinic (ICC), which is a partnership between Pathways to Housing PA, a housing-first agency and Project HOME Healthcare Services (PHHS), a Federally Qualified Health Center (23, 24). The ICC includes addiction and primary care physicians, psychiatrists, social workers, and support staff. The clinic serves a steady population of patients with OUD for ongoing primary care and MOUD.

The evaluation is an initial observational evaluation with the potential to replicate in similar primary care settings. To improve the evaluation’s inferential strength, we hope to compare results with affiliated, partnered, or otherwise interested clinics concurrently treating OUD while not yet adopting MOUD 2.0. Examples of clinics for comparison could include other sites of PHHS. Since these clinics have not yet fully crossed over to MOUD 2.0, they may be potential sites for intra- and multi-site stepped wedge randomized clinical trials of MOUD 2.0, pending initial results of the implementation evaluation. A stepped wedge design or other randomized clinical trial would also avoid the limitations of observational studies discussed further below (25).

Data will be de-identified prior to statistical analysis and reporting. Therefore, the Philadelphia Department of Public Health and Thomas Jefferson University IRB provided the evaluation exempt status.

### Setting and population

Given the clinic’s size and an accrual time of 2 years including before and after algorithm implementation, we anticipate approximately 100 participants for this evaluation. Participants will be individuals age 18 or older with current or past experience of homelessness and a diagnosis of OUD. All participants are patients at the ICC. Demographic information will be reported. The sample for comparing adherence will be restricted to participants already enrolled in the ICC for management of OUD prior to the algorithm implementation, measuring adherence at 6 months prior to implementation and 6 months after. Survival curves will be drawn from all aggregated patients in MOUD programs at 6 months pre-implementation to implementation or dropout, and from 6-12 months post-implementation or dropout.

### Evaluation procedures, data sources, and variable abstraction

Evaluation procedures are limited to review of medical records that will already be in existence. The evaluation will include participants who received treatment for OUD between 6/1/2022 and 1/1/2024 and a random sample of patients without OUD within the same date range. Follow up information will be included on all participants up until 1/1/2025.

Data will be collected through retrospective chart review of the ICC’s electronic medical record. Corresponding to the listed date ranges, these data will be measured at six months before implementation of MOUD 2.0 and six months after implementation. Data will then be de-identified and sent to team members for analysis.

Demographics and medical history will have already been included in the electronic medical record from the standard intake forms used to begin patient encounters. Variables to be abstracted from the medical record include:

1. Demographic information: age, sex, racial self-identification.
2. Adherence to MOUD (categorical, based on positive buprenorphine toxicology or concurrent negative opioid and buprenorphine toxicology) at collection time point.
3. Use of opioids during treatment periods.
4. Months in MOUD program, up to 1 year.
5. Month/year of MOUD visits.
6. Month/year of LAIB injection.
7. Results of urine drug screens while in program.
8. Treatment Effectiveness Assessment Score (21).
9. Results of HIV/HCV screening labs while in program.
10. Dates, dosage, and quantity of buprenorphine prescriptions.

Adherence is difficult to determine from self-report in patients with OUD due to factors like diversion, stigma, and bias (26, 27). Previously published non-LAIB algorithms did not incorporate objective measures besides self-report. To improve accuracy in treatment, adherence will be determined based on toxicology as described in the algorithm.

The approach to urine drug screening was non-standard in the months prior to implementation and continues to reflect the harm reduction approach to treatment. Therefore, adherence will be operationalized as having a minimum of 3 buprenorphine- and norbuprenorphine-positive (“appropriate”) drug screens over a 6-month period regardless of substance use. Each screen should be at least 2 weeks apart within this time period. In each phase of the evaluation, participants will be dichotomized into “adherent” vs “non-adherent” using the above definition.

### MOUD 2.0 algorithm

**Figure 1:**
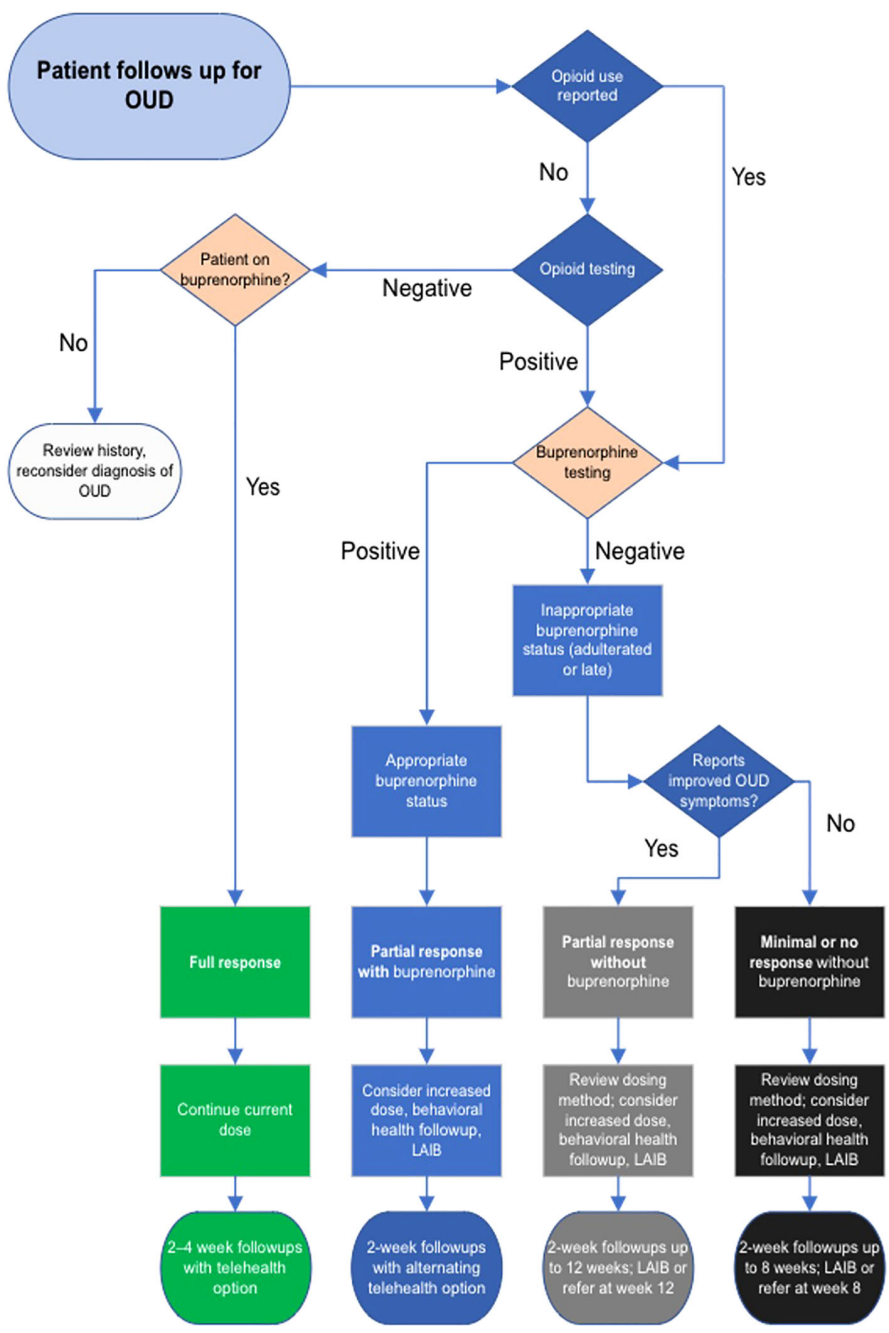
Clinical decision algorithm for treating opioid use disorder with sublingual and injectable buprenorphine.

The MOUD 2.0 algorithm was implemented at the clinic-wide level in January 2023. Staff at the ICC, Pathways to Housing PA, and PHHS developed the algorithm for use at follow-up visits for OUD after the initial diagnosis visit (see **Table 1** and below **Figure 1**). The algorithm has 4-, 8-, and 12-week decision points to guide treatment for patients with varying responses to sublingual and injectable buprenorphine.

**Table 1:** MOUD 2.0 status and algorithm responses.

Status	Symptoms	Opioid Use	Opioid level	BUP adherence	BUP level	Plan	Visit interval
Full response	Improved	None	Negative	Full	Appropriate	Continue current dose	2 weeks in-person alt 2 week TH option OR 4 weeks in-person
Partial response with BUP	Improved	Some	Positive	Partial	Appropriate	Consider: Increase dose Regular BH f/u LAIB	2 weeks in-person alt 2 week TH option
Partial response without BUP	Improved	Some	Positive	Partial	Negative OR Adulterated OR Late	Review dosing method Consider: Increase dose Regular BH f/u LAIB	2 weeks in-person for up to 12 weeks LAIB or refer at week 12
Minimal or nonresponse without BUP	No improvement	Some	Positive	Partial	Negative OR Adulterated OR Late	Review dosing method Consider: Increase dose Regular BH f/u LAIB	2 weeks in-person for up to 8 weeks LAIB or refer at week 8
Reconsider diagnosis of OUD	Any	Any	Negative	Any	Negative OR Adulterated OR Late	Review hx/reconsider diagnosis of OUD	2 weeks in person Discharge or refer at 4 weeks

“Appropriate” indicates buprenorphine and norbuprenorphine detected in urine without contaminants.

*BUP* buprenorphine, *BH f/u* behavioral health follow up, *LAIB* long-acting injectable buprenorphine, *OUD* opioid use disorder, *alt TH* alternating telehealth.

Patients are clinically categorized as “full response,” “partial response with buprenorphine,” “partial response without buprenorphine,” “minimal or nonresponsive without buprenorphine,” and “reconsider diagnosis of OUD.” Note that “full” or “partial response” in the algorithm refers not to adherence or non-adherent outcomes but are clinical categories of physiological response informing further treatment decisions. Physiological response is important *within* the algorithm but is not an outcome measure for successful algorithm implementation.

Depending on the response, the algorithm recommends a plan and follow-up interval. Categories are based on subjective improvement of OUD symptoms in response to buprenorphine, in combination with objective measures of MOUD adherence. Symptoms include cravings, withdrawals, and interference with other activities and functioning with reference to DSM-5 diagnostic criteria for OUD, and are subjectively reported by the patient as improved, the same, or worse compared to the previous visit.

Because a major goal of MOUD 2.0 is increasing buprenorphine adherence, toxicology is included as a clinical data point to guide decision-making (28). Patients subjectively report non-prescribed opioid use, which is confirmed by urine or saliva drug screen including fentanyl and other opioids. Urine testing for appropriate levels of buprenorphine and metabolites confirms buprenorphine adherence. The combination of subjective patient report, urine opioid screen, and urine buprenorphine testing results in the patient’s status that the algorithm assigns (see **Table 1**).

For example, “full response” patients report improved symptoms, demonstrate appropriate qualitative levels of buprenorphine and its metabolite norbuprenorphine, and show no evidence of opioid use on urine screen. Improved symptoms include findings such as fewer cravings, better functioning in everyday activities, and less time spent for obtaining opioids, etc. (19). These patients continue their regimen of buprenorphine and clinic visits every 2-4 weeks, at shared discretion. Buprenorphine dose will remain unchanged as their current dose and delivery route of buprenorphine appear to be working well.

Patients with partial responses may have some improvement of OUD symptoms but still demonstrate some use of opioids by self-report or urine screen. They are either not being sufficiently treated with buprenorphine or are non-adherent to medication. If urine shows buprenorphine and norbuprenorphine, the dose of sublingual buprenorphine may be too low, or the patient would benefit from LAIB. If urine is negative for buprenorphine and the patient demonstrates opioid use, treatment should progress with redosing or LAIB within a 12-week timeline. This timeline ensures the patient benefits from treatment while limiting medication diversion and other negative social effects, as further MOUD treatment for non-adherent patients in primary care may cause net harm. Patients with little or no response to treatment, i.e. without OUD symptom improvement and without adherence to buprenorphine treatment, are likewise accounted for.

LAIB provides a definitive treatment for suitable patients within the various visit timelines, and it can also be used earlier in treatment for motivated patients. Closer follow-up benefits patients who need dose adjustments. The clinician using the algorithm adjusts buprenorphine dosing, recommends injectable buprenorphine, or refers the patient to another program as indicated.

### Algorithm development, staffing, and funding in a low-barrier setting

The ICC, the setting for the development of the algorithm, provides medical, behavioral health, substance use treatment, and housing services. Since 2016, the clinic’s services have included low-barrier, primary care-based MOUD. The low-barrier approach was first described with methadone maintenance therapy (29) and has been extended to buprenorphine treatment (30). This approach includes same-day treatment entry, harm reduction, flexibility in appointments and prescribing, and combining services in one location.

The ICC participates in the Pathways housing-first model, which offers immediate access to one-bedroom scattered-site apartments with intensive support services for people with experiences of chronic homelessness. All participants have a diagnosis of a serious mental illness or substance use disorder. Many participants were previously living unsheltered in the Kensington neighborhood of Philadelphia, a neighborhood often called the “epicenter of the opioid crisis,” with high levels of OUD, homelessness, food insecurity, and early mortality (31–33). The ICC continued increasing the number of people treated for OUD and modified its services throughout the COVID pandemic. As multiple other low-barrier MOUD clinics have reported, the ICC MOUD program made several intentional adjustments to MOUD care delivery to facilitate access despite disruptions. Such adjustments included: 1. extended visit intervals of up to 2 weeks during the initiation and stabilization phase of treatment with telehealth options, and, 2. a goal of monthly urine drug screening for all patients, but tolerating longer intervals without penalty.

This approach was successful in preserving access and retention in services from March 2020–March 2022. However, as an unintended consequence, the clinical team expressed increasing concerns regarding non-adherence to buprenorphine and lack of consistency in programmatic response, calling into question the program’s overall effectiveness. These concerns and the recent introduction of LAIB to clinical practice necessitated the algorithm’s development and an implementation evaluation to assess the algorithm’s effectiveness. Following the program’s philosophy of participation, the algorithm and implementation process were refined over a series of meetings with the medical team, separate meetings with the Center of Excellence care management team, meetings with Pathways leadership and Project HOME leadership, and focus groups with current program patients.

The ICC partners with several other organizations to receive funding from the Pennsylvania Department of Human Services as an OUD treatment Center of Excellence. The Center of Excellence program provides funding for care managers at its partner sites. While the ICC’s operations are funded by the Center of Excellence program, this evaluation does not receive additional funding for its development and execution.

### Data analysis

Baseline and demographic characteristics will be summarized with descriptive statistics such as means and standard deviations for continuous variables such as age and percentages for categorical variables such as race.

To accommodate the need for OUD treatment centers to have fair and ethical treatment of patients at the clinic-wide level, we will use patients as their own controls and assess six-month adherence before and after program implementation. The evaluation will follow the same patients, either adherent or non-adherent at six months before algorithm implementation, by chart review to see if they crossed over to the opposite outcome six months after implementation. We will present adherence at endpoints six months prior to and six months after MOUD 2.0 implementation as proportions. We will qualitatively compare the proportion of adherence six months prior, i.e. adherence in 2022, to the proportion six months after. Then, a chi-squared test will compare discordant pairs, i.e. patients who became newly adherent after MOUD 2.0 and patients who became newly non-adherent.

Because we are treating adherence as a binary outcome, we will use McNemar’s test to determine differences in adherence before and after implementation, with each individual patient before and after implementation as a paired sample. The Chi-squared test statistic for McNemar’s test takes the form:


$$\chi^{2}=\frac{{\left(b-c\right)}^{2}}{b+c}$$

where χ^2^ follows the chi-squared distribution with 1 degree of freedom, *b* represents the patients who were adherent before and were not adherent after, and *c* represents the patients who were not adherent before and were adherent after.

Finally, we will present unadjusted six-month survival curves for retention before and after implementation. While comparing unadjusted survival curves before and after MOUD program implementation has potential for survival bias (i.e., individuals need to “survive” to program implementation in order to be considered for post-implementation calculations), this comparison is given to provide a general sense of how MOUD 2.0 might affect the clinic overall and not to predict explicit dropout rates. If there is a significant number of patients not retained, descriptive statistics for this group will be reported to characterize factors associated with dropout. Secondary outcomes such as receipt of LAIB and use of opioids and of other substances during the evaluation periods will be analyzed with linear regression models for descriptive purposes. These primary and secondary outcomes may also be used for a future stepped wedge analysis in other clinics that could use the algorithm prospectively.

### Sample size and power

Considering the primary outcomes of six-month adherence, crossover, and six-month survival in the program, a sample size of 86 was calculated based on the standard sample-size formula for the proportional-hazards regression model (34). Since the evaluation is a clinic-wide retrospective implementation evaluation, it is beneficial to include as many eligible patients as receive MOUD treatment at the clinic. Therefore, a sample size of approximately 100 based on the typical clinic census will be used as a sample size of convenience as well as for improved power.

### Evaluation status

The MOUD 2.0 clinical algorithm was developed during the COVID-19 pandemic and implemented in the ICC in January 2023. The protocol for a retrospective implementation evaluation of the algorithm was given notice of IRB exemption in February 2023. As the evaluation involves retrospective chart review, no patients were actively recruited during this time period, but new patients continue to enroll or elect to discontinue treatment, and follow up of patients for this evaluation will be completed in early 2025. This manuscript was based on protocol version 3.

## Discussion

This is the first clinical algorithm we are aware of that incorporates long-acting injectable buprenorphine in treatment of opioid use disorder. LAIB adds an additional layer to a clinic’s strategy for improving communication and adherence between clinicians and patients. For primary care settings where monitored treatments are unfeasible, LAIB provides assurance that treatment is being delivered to its intended recipients while reducing the number of visits and improving rapport between staff and patients. Primary care settings may lack guidance on incorporating a new administration route such as LAIB. Thus, this algorithm positions LAIB among early treatment options as well as part of a stepwise progression.

Furthermore, this algorithm builds on strengths of previously published algorithms (17, 35) and offers a way to assess their effectiveness indirectly. Previous algorithms were not evaluated for their effects on patient populations using statistical methods. Therefore, there are limited data on the effects of introducing more standardized treatment options for OUD in primary care. This evaluation will add to MOUD options in primary care while serving as a template for evaluating new and existing protocols. This and future studies could also provide settings for validating screening tools for measurement-based care, by combining these screens with toxicology results.

Because of the high morbidity and mortality of OUD and the marginalized patient population, designing an ethical observational study or clinical trial can be difficult, which could possibly have limited previously published algorithms from being evaluated. This evaluation addresses such concerns by controlling by chronology and including all eligible patients in clinic-wide implementation of the algorithm. Including all patients not only promotes ethical treatment to produce satisfactory outcomes, but also removes a barrier to treatment that new treatment strategies may introduce: A new intervention such as LAIB can lead to some patients perceiving unfairness if other patients receive the intervention first, or they may feel they are being singled out for more intrusive followup if offered the treatment before others. Situating LAIB in a clinic-wide pathway promotes transparency with patients and should reduce the attrition that can be associated with introducing a new treatment strategy.

A limitation of this evaluation includes the necessity of assigning categories in the algorithm and evaluation, at the expense of nuance. The algorithm sorts patients into full, partial, and no response groups, but the ways patients benefit from MOUD can be subtle and vary from day to day. For example, a patient may benefit from buprenorphine one week and then experience a social stressor the next week resulting in diversion of buprenorphine and return to use. The narratives of such experiences are important, yet the clinical team must balance fairness with attentiveness to individual needs, as the algorithm categories demonstrate. For the sake of statistical analysis, the evaluation protocol cannot fully account for these variations when assigning binary outcomes. There are also inherent limitations to observational studies such as vulnerability to confounding, as this is not a blinded randomized clinical trial. These concerns can be mitigated by replicating the evaluation across multiple different primary care settings. As noted, after an initial implementation evaluation, a stepped wedge randomized clinical trial design could be used in similar clinics. The goals of this initial evaluation are different from those of a clinical trial, as the questions are whether the algorithm is feasible to implement and whether it benefits the MOUD clinical setting overall, not whether each individual administration of a medication has the desired outcome. Complete elimination of confounders is thus not necessary or desirable in this evaluation.

A final strength of this evaluation is the design of the algorithm in consultation with many representatives of the community, including patients (36). This algorithm was unique as it originated not only from the medical team but also through meetings with clinic management staff, leadership, and focus groups with patients. The algorithm’s development was consistent with a principle of transparency that the algorithm was intended to honor. In making this algorithm and its evaluation protocol available, greater transparency becomes available for assessing whether MOUD strategies based on this principle are effective in other primary care settings.

## Conclusion

Clinicians need the best available tools for treating OUD in primary care settings, where OUD is most likely to be encountered but also where clinical decision support is still developing. This primary care clinical support algorithm is unique in including injectable buprenorphine and urine toxicology for treating OUD. The algorithm draws from previous algorithms and feedback from clinic staff and patients to promote fairness and transparency. The goal of implementing the algorithm was to improve adherence and retention, thereby expanding access to quality care. In keeping with this principle of transparency for MOUD 2.0, we have included an algorithm evaluation protocol to be implemented in a primary care setting with a high volume of OUD and which may be replicated in similar settings.
